# Ventromedial Hypothalamus and the Generation of Aggression

**DOI:** 10.3389/fnsys.2017.00094

**Published:** 2017-12-20

**Authors:** Yoshiko Hashikawa, Koichi Hashikawa, Annegret L. Falkner, Dayu Lin

**Affiliations:** ^1^Neuroscience Institute, New York University School of Medicine, New York University, New York, NY, United States; ^2^Department of Psychiatry, New York University School of Medicine, New York University, New York, NY, United States; ^3^Center for Neural Science, New York University, New York, NY, United States

**Keywords:** VMHvl, aggression, mouse, neural activity, neuromodulation

## Abstract

Aggression is a costly behavior, sometimes with severe consequences including death. Yet aggression is prevalent across animal species ranging from insects to humans, demonstrating its essential role in the survival of individuals and groups. The question of how the brain decides when to generate this costly behavior has intrigued neuroscientists for over a century and has led to the identification of relevant neural substrates. Various lesion and electric stimulation experiments have revealed that the hypothalamus, an ancient structure situated deep in the brain, is essential for expressing aggressive behaviors. More recently, studies using precise circuit manipulation tools have identified a small subnucleus in the medial hypothalamus, the ventrolateral part of the ventromedial hypothalamus (VMHvl), as a key structure for driving both aggression and aggression-seeking behaviors. Here, we provide an updated summary of the evidence that supports a role of the VMHvl in aggressive behaviors. We will consider our recent findings detailing the physiological response properties of populations of VMHvl cells during aggressive behaviors and provide new understanding regarding the role of the VMHvl embedded within the larger whole-brain circuit for social sensation and action.

## An essential role for the VMHvl in aggressive behaviors

Aggression is an innate social behavior essential for resource competition, settling disputes, defense, and protecting kin. It is a prevalent behavior across many species, including humans, and in a variety of species including cats, rats, chickens, and monkeys, electrical stimulation studies have demonstrated a causal role of the medial hypothalamus in expressing aggressive behaviors (Putkonen, 1966; Siegel and Skog, 1970; Lipp and Hunsperger, 1978; Kruk et al., 1979, 1983; Lammers et al., 1988; Siegel and Pott, 1988; Siegel et al., 1999; Halasz et al., 2002; Nelson and Trainor, 2007). Recent studies using more precise functional manipulation tools in mice have identified the ventrolateral part of the ventromedial hypothalamus (VMHvl), a small subnucleus in the medial hypothalamus, as a key region to drive inter-male aggression (Table 1). Silencing this area abolishes naturally occurring inter-male attack whereas optogenetic activation of the VMHvl but not its surrounding regions promotes the attack of suboptimal targets, including females and inanimate objects (Lin et al., 2011). The VMHvl is highly enriched in hormone receptors, including estrogen receptor alpha (Esr1) and progesterone receptor (PR) which co-express in nearly 100% of cells. Several converging studies have recently demonstrated that these hormone receptor-expressing cells appear to be key populations for mediating aggression in both males and females (Yang et al., 2013, 2017; Lee et al., 2014; Hashikawa et al., 2017). Killing PR+ cells or inhibition of the Esr1+ cells suppressed naturally occurring aggression (Yang et al., 2013; Lee et al., 2014; Hashikawa et al., 2017). Conversely, optogenetic activation of the VMHvl Esr1+ cells elicited immediate attack and pharmacogenetic activation of the PR+ cells increases the frequency of attack (Lee et al., 2014; Hashikawa et al., 2017; Yang et al., 2017). Furthermore, the aggression-promoting effects of activating VMHvl PR+ cells were also observed in castrated males and also in males with defective olfactory inputs, suggesting that the VMHvl activation can “override” normal hormone and sensory requirements for aggression (Yang et al., 2017).

**Table 1:** VMHvl is essential for aggression.

	**Manipulation**	**Sex**	**Population**	**Test**	**Behavior**	**References**
Gain of function	ChR (20 ms, 20 Hz)	Male	VMHvl	R-I test	Attack	Lin et al., 2011; Falkner et al., 2016
	ChR2 (20 ms, 20 Hz), high intensity	Male	VMHvl Esr1+	R-I test	Attack	Lee et al., 2014
	ChR (20 ms, 20 Hz), low intensity	Male	VMHvl Esr1+	R-I test	Close investigation, mounting	Lee et al., 2014
	DREADDq (CNO, i.p. injection)	Male	VMHvl PR+	R-I test	Increase attack frequency	Yang et al., 2017
	ChR (20 ms, 20 Hz)	Female	VMHvl Esr1+	R-I test	Attack	Hashikawa et al., 2017
	ChR2 (20 ms, 5 Hz)	Male	VMHvl	SIA test	Shorten poke latency	Falkner et al., 2016
Loss of function	GluCL (IVM, i.p. injection)	Male	VMHvl	R-I test	Reduce attack	Lin et al., 2011
	DREADDi (CNO, i.p. injection)	Male	VMHvl	R-I test	Reduce attack	Falkner et al., 2016
	NpRH (continuous light)	Male	VMHvl Esr1+	R-I test	Block attack	Lee et al., 2014
	Caspase 3 (ablation)	Male	VMHvl PR+	R-I test	Reduce attack	Yang et al., 2013
	DREADDi (CNO, i.p. injection)	Female	VMHvl Esr1+	R-I test	Reduce attack	Hashikawa et al., 2017
	DREADDi (CNO, i.p. injection)	Male	VMHvl	SIA test	Reduce poke rate	Falkner et al., 2016

*A summary of functional manipulation experiments that support an essential role of the VMHvl in conspecific aggression*.

**Figure 1:**
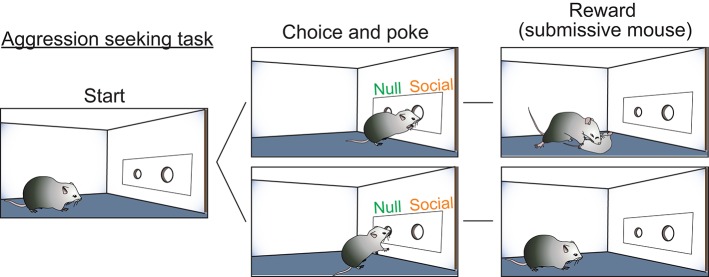
Self-initiated aggression seeking task is utilized to study appetitive phase of aggression. Schematic illustration of the self-initiated aggression seeking task. By poking the social poke, subjects gain access to a submissive intruder and attack.

While these studies clearly implicated hormone-receptive populations in the generation of aggression, the role of the VMHvl in this behavior remained unclear. Is the VMHvl simply an “attack generator” or does it also promote flexible aggression seeking behaviors that lead to attack? As evidence for aggression-seeking behavior, it has been observed behaviorally from fish to primates that certain individuals will develop a strong preference for the context where they attacked a conspecific (Meisel and Joppa, 1994; Martinez et al., 1995; Golden et al., 2016) and will voluntarily seek the opportunity to attack a conspecific (Thompson, 1963; Cherek et al., 1973; Turnboug and Lloyd, 1973; Fish et al., 2002, 2005; May and Kennedy, 2009; Mitani et al., 2010; Golden et al., 2017). To test the role of the VMHvl in aggression seeking, we designed a self-initiated aggression-seeking (SIA) task during which the animals learn to voluntarily nose poke to gain access to a weaker male intruder (Falkner et al., 2016). Over weeks of training, the majority of trained males exhibited task-dependent learning. These animals demonstrated a clear preference for the nose port associated with the weak intruder, poked it repeatedly and attacked the intruder immediately after its introduction (Figure 1). We found that low-level optogenetic activation of the VMHvl cells reliably reduced the animals' latency to nose poke for an opportunity to attack. Conversely, inhibiting the VMHvl suppressed nose poking for the chance to attack but not for water reward, demonstrating a role for the VMHvl in aggression seeking in addition to attack (Falkner et al., 2016).

It is important to note that this area is not exclusively implicated in aggressive behaviors. In addition to aggression, the VMHvl is also well-known for its essential role in female sexual behaviors and to a lesser extent, in male sexual behaviors (Pfaff and Sakuma, 1979a,b; Yang et al., 2013; Lee et al., 2014; Hashikawa et al., 2017). Furthermore, the VMHvl may also mediate behaviors against an aggressor: immediate early gene mapping experiments have revealed strong activation of the area in subordinate animals after social defeat (Kollack-Walker et al., 1997; Motta et al., 2009; Pan et al., 2010; Silva et al., 2013) and re-activation of the defeat induced Fos population in the VMHvl elicits fearful responses (Sakurai et al., 2016). Thus, the VMHvl likely mediates multiple social behaviors and future studies will need to address how factors including social experience, hormonal state, and behavioral context influence which behaviors are generated.

## The encoding of aggression-related information in the VMHvl

**Figure 2:**
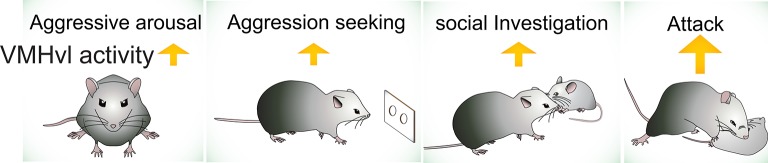
The activity of VMHvl increases during social investigation, aggression seeking and attack, and when the animal is at a heightened aggressive state.

Among identified aggression-related circuits in the brain, the VMHvl is currently the best understood area, due in large part to our characterization of the response properties of neurons during social behaviors. We have performed extensive electrophysiological recording in the VMHvl in freely-moving, socially-interacting animals (Lin et al., 2011; Falkner et al., 2014, 2016; Wong et al., 2016). Based on these data, we propose that VMHvl cells encode at least three features of aggression-related information: (1) the overall aggressive state of the animal (*motivation*); (2) the detection of aggression-provoking sensory cues (*sensation*); and (3) the initiation and execution of attack and aggression-seeking behaviors (*action*) (Figure 2). We hypothesize that the aggressive state is encoded as the baseline spiking activity of the VMHvl cells whereas the sensory information and the motor actions are encoded by acute changes in VMHvl activity.

### Aggressive arousal state

Upon the intruder introduction, VMHvl activity quickly increases and is maintained at a high level. This elevation in spontaneous activity is not associated with any particular behavior and can account for ~50% of the total VMHvl firing rate increase (Lin et al., 2011; Falkner et al., 2016; Hashikawa et al., 2017). In addition, VMHvl activity can remain at an elevated state for minutes after the intruder removal (Falkner et al., 2014). Increased VMHvl activity after intruder removal coincides with a heightened aggressive state. If a second intruder is presented shortly after removing the first intruder, both the latency to attack decreases and the probability of attack increases (Potegal et al., 1996). As further demonstration of this, in tests of the self-initiated aggression task, after the animal learned the task contingency, similar sustained increase in spiking activity was observed after the nose poking apparatus was introduced into the cage of the intruder but before any nose poking or attack (Unpublished data by Annegret Falkner). Consistent with a heightened aggressive state of the animal during the self-initiated aggression task, the resident male typically attacks the intruder immediately after the intruder becomes available. These electrophysiological and behavioral observations lead us to hypothesize that the spontaneous activity of the VMHvl signals the general aggressive state of the animal.

This property may be common to the hypothalamus and associated neural circuit. Other internal states such as hunger and thirst have also been shown to be encoded by the spontaneous activity of specific populations of neurons. For example, AgRP neurons in the arcuate nucleus promote feeding and food seeking when artificially activated and these neurons show sustained increase in neural activity in food deprived animals (Aponte et al., 2011; Krashes et al., 2011; Betley et al., 2015; Chen et al., 2015). Similarly, subfornical organ (SFO) neurons can promote drinking and seeking for water when artificially activated and show sustained increase in spontaneous activity in water deprived animals (Oka et al., 2015; Zimmerman et al., 2016). While in those cases, changes in spontaneous activity are caused by changes in interoceptive physiological signal, in our experimental condition, elevation in VMHvl spontaneous activity is triggered by external cues, such as conspecific intruders or aggression-associated object. However, an aggressive internal state can also be signaled internally through experience. For example, male mice that repeatedly encounter a male intruder at the same time of the day learn to anticipate the fighting as indicated by their increase in heart rate and body temperature prior to the scheduled fighting time (Tornatzky et al., 1998). Whether the internally generated aggressive state is also accompanied and/or caused by an increase in spontaneous VMHvl activity remains to be confirmed.

### Aggression-provoking olfactory cues

VMHvl cells also robustly respond to olfactory cues associated with aggression-provoking stimuli (Falkner et al., 2014). During investigation of male intruders, male-responsive VMHvl cells acutely increase activity from an already elevated baseline. Moreover, when recorded males investigated urine from the intruder, VMHvl activity also increased in a subpopulation of cells (Lin et al., 2011; Falkner et al., 2014). Urine is a rich source of pheromones and carries important information regarding the sex, physiological, and social status of the animal (Dulac and Torello, 2003). Several volatiles and major urinary proteins have been identified in male mouse urine that can promote aggression when applied onto the intruder mouse (Novotny et al., 1985; Chamero et al., 2007). As a demonstration of this, castrated male intruders, whose urine contains fewer critical volatiles are less likely to be attacked by resident male mice (Mugford and Nowell, 1970) and urine from castrated male mice generate less activity from VMHvl cells in comparison to intact male mouse urine (Falkner et al., 2014). Male mice also rarely attack females, and female urine increases activity in only a small subpopulation of VMHvl neurons in aggressive males (Falkner et al., 2014).

### Aggressive actions

During free social interactions, the most prominent activity increase observed in VMHvl cells is from attack itself. Activity in responsive cells rises prior to attack (~1 s), peaks at the onset of attack, sustains throughout the duration of attack, and quickly drops to the baseline level at the behavioral offset (Falkner et al., 2014). Importantly, movements unrelated to attacking do not correlate with or predict VMHvl activity (Falkner et al., 2014). In addition, several complimentary lines of evidence suggest that this increased activity during attack cannot be simply accounted by the increased activity from sensory cues. First, the response during attack is higher than the responses during investigation that precedes attack (Falkner et al., 2014; Hashikawa et al., 2017). Second, when we compare the isolated aggression trials (attack not preceded or followed by attack) and investigation trials (investigation not followed or preceded by attack), we find that attack responses are significantly higher during these isolated attack trials (Hashikawa et al., 2017). Third, in a linear regression model that explores the relationship between various behavioral parameters and VMHvl cell activity, we found that the inclusion of a “latency to attack” parameter can significantly improve model fit in a subpopulation of cells beyond what can be accounted for considering the distance between animals (an estimate of sensory input) and the movement velocity of the animal. This data quantitatively supports the hypothesis that the VMHvl activity carries information regarding the initiation of attack independently of these external cues (Falkner et al., 2014). Furthermore, using the self-initiated aggression seeking task as a way to separate actions associated with seeking and attack, we found that a subset of VMHvl cells increase spiking activity prior to and during nose poking once the animal learned the association between nose poking and future attack (Falkner et al., 2016). This shows that the VMHvl cells not only signal the initiation of physical attack but also flexible learned actions that lead to future attack.

Aggressive state, aggression-provoking sensory cues, and aggressive actions are encoded by highly overlapping populations of VMHvl cells. Approximately half of the VMHvl cells that respond during attack and investigation showed sustained increase in spontaneous activity in the presence of intruder (Lin et al., 2011). The cell responses during attack and social investigation are significantly correlated (Falkner et al., 2014). Approximately 80% of cells that increase activity during aggression seeking also increase activity during attack (Falkner et al., 2016). Thus, the activity of a single VMHvl cell at any given moment can encode multiple aspects of aggression-related information and these signals may be linearly summed to create the observed response profile.

Other hypothalamic subregions may encode similar multifaceted responses for other social behaviors. One well-studied example of this is the responses of cells in the medial preoptic area (MPOA) during sexual behaviors. *In vivo* extracellular recording from the MPOA in freely moving rats revealed that MPOA cells show sustained increase in activity after female introduction as well as acute increase in activity during female investigation and specific sexual actions, such as pursuing, mounting, and intromission (Oomura et al., 1983; Horio et al., 1986; Shimura et al., 1994). Additionally, an acute increase in the MPOA cell activity was observed every time when the male operated a lever to bring a female closer to him (Oomura et al., 1988). Thus, similar to VMHvl activity change during aggression, the MPOA cells signal the animal's sexual arousal, detection of sexual arousal cues, and specific preparatory and consummatory sexual actions. Taken together, we speculate that the same general coding principles are employed by hypothalamic networks to represent sensory, arousal (motivation) and action-related information essential for social behaviors.

### Experience-dependent changes in VMHvl cell responses

Mounting evidence now suggests that the response properties of VMHvl neurons are not fixed as would be in a “hard-wired” innate circuit, but instead are constantly updated. Recently, Remedios et al. used microendoscopic calcium imaging to examine changes in VMHvl cell activity over days as the recorded males encountered male and female intruders and obtained social experience (Remedios et al., 2017). While the VMHvl responses during male and female investigation heavily overlap initially in naïve unexperienced males, brief sexual experience with females caused significant divergence of the VMHvl cell responses. Importantly, this divergence only occurred when animals started to show mounting and fighting, suggesting a potential causal link between the change in neural responses and the selection of appropriate social actions. Additionally, we also observed changes in VMHvl activity over the training of the self-initiated aggression seeking task (Falkner et al., 2016). In early training phase before the animals made the association between nose poking and the opportunity to attack, little response of VMHvl cells during poking was found. As the training went on and animals successfully learned the task contingency, VMHvl cells showed clear activity increase prior to, during and after the nose poking, supporting the capacity of VMHvl cells to change responses with experience.

Experience-dependent changes in VMHvl activity also appear to alter the efficacy of attack initiation. In early electric stimulation experiments in rats, it was found consistently that repeated attack induced by electric stimulation reduced the amount of current required to elicit attack in subsequent testing days (Kruk, 2014). This threshold-lowering effect requires the attack itself (i.e., it cannot be induced via stimulation in isolation) (Kruk et al., 1979; Kruk, 2014). More recently, Yang et al. examined the reliability of VMHvl activation-induced aggression in animals with different experience and under different testing environment (Yang et al., 2017). They expressed an engineered ligand (CNO) gated Gq coupled receptor, DREADDq, in the VMHvl and examined the CNO injection induced attack in animals that are single- vs. group-housed and in home territory vs. foreign territory. They found that while attack can be reliably chemogenetically induced in single-housed mice in both home and foreign territory, this same manipulation is only effective in inducing attack in group housed males when tested in home territory. The difference in attack induction efficacy between single-housed and group-housed animals is likely due to either to differences in the physiological properties of the VMHvl itself, or to changes in its inputs. In the VMHvl itself, intrinsic excitability may be higher in singly-housed animals than group-housed animals and VMHvl neurons in singly-housed animals are likely to fire more readily when activated using DREADDq. Alternatively, cues related to foreign territory may cause a stronger suppression of VMHvl cells in group-housed animals and prevent the cells from spiking in a foreign territory. Consistent with this second idea, a high efficacy of stimulation-evoked attack can be “revealed” in group-housed animals in a foreign environment when the olfactory inputs were blocked (Yang et al., 2017). Future experiments that examine the physiological and synaptic properties of VMHvl cells from animals with different experience and aggression level will help elucidate the dynamic range of VMHvl cell physiological properties and whether hyper-excitability of VMHvl cells could also be related to the exaggerated aggression observed under certain pathological conditions in human patients and animal models, such as autism, borderline personality disorder, and posttraumatic stress disorder (Kanne and Mazurek, 2011; Jiang-Xie et al., 2014; Wells et al., 2016).

## Molecular and circuit mechanisms for driving aggression-relevant VMHvl activity

### Circuits for olfactory input

**Figure 3:**
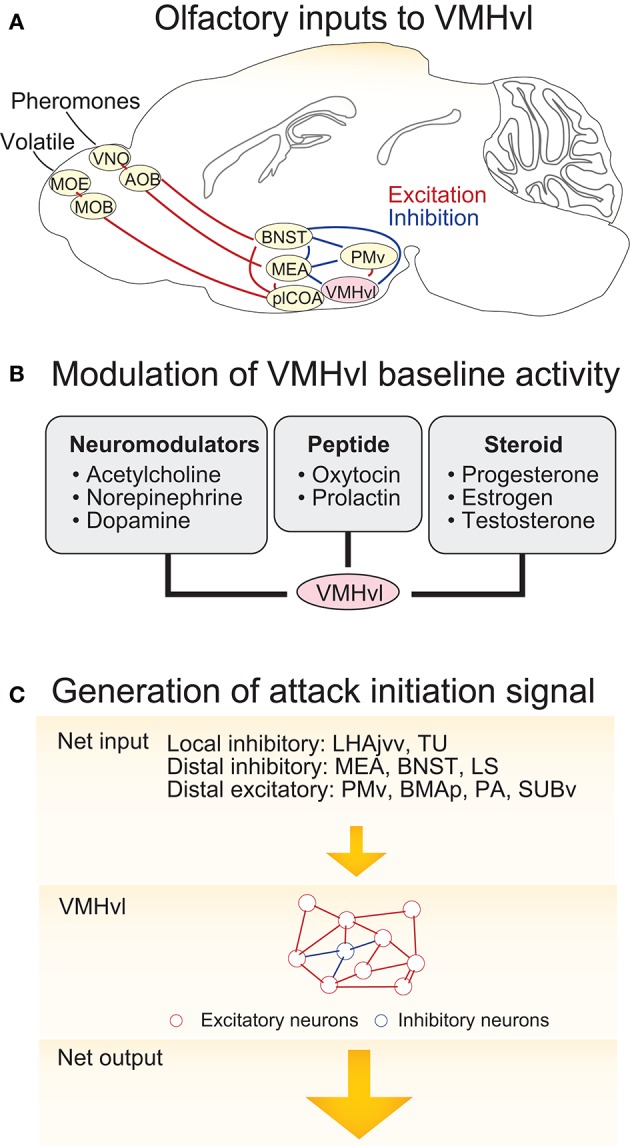
Circuit mechanisms underlying VMHvl activity change during agonistic encounters. **(A)** The neural circuits upstream of the VMHvl that relay olfactory information. **(B)** Neuromodulators, neuropeptides, and neurosteroids that could potentially generate increased spontaneous activity in the VMHvl and cause sustained aggressive state. **(C)** Schematics illustrating a model responsible for activity increase at the VMHvl during attack initiation. In the model, inputs from the upstream regions bring the VMHvl activity to a threshold and then the recurrent excitatory network within the VMHvl quickly amplifies the signal to initiate attack and maintain it throughout the attack. OE, olfactory epithelium; VNO, vomeronasal organ; MOB, main olfactory bulb; AOB, accessory olfactory bulb; BNST, bed nucleus of stria terminalis; MEA, medial amygdala; plCOA, posterolateral cortical amygdala; PMv, ventral premammillary nucleus; VMHvl, ventromedial hypothalamus ventrolateral part. LHAjvv, lateral hypothalamic area, juxtaventromedial region, ventral zone; TU, tuberal nucleus; LS, lateral septum; BMAp, posterior basomedial nucleus; PA, posterior amygdala; SUBv, ventral subiculum.

The VMHvl responses that signal aggressive state, sensory detection, and aggressive action may result from independent sources of activity which include both sensory inputs and changes in neuromodulatory tone. Among these potential molecular and synaptic drivers, the VMHvl responses to the olfactory cues are the best understood since pathways that convey olfactory information have been previously identified (Figure 3A). The VMHvl receives converging volatile and pheromone information from the medial amygdala (MEA) and bed nucleus of stria terminals (BNST) (Canteras et al., 1994). Volatile information passes through the main olfactory epithelium, main olfactory bulb, posterolateral cortical amygdala (plCOA) and arrives at the MEA and BNST whereas pheromone information is relayed through the vomeronasal organ (VNO), accessory olfactory bulb (AOB) and then also reaches the MEA and BNST (Hashikawa et al., 2016). Recent tracing studies also suggest that MOB mitral and tufted cells may directly project to the MEA (Pro-Sistiaga et al., 2007; Kang et al., 2009, 2011). Regardless, converged volatile and pheromone information at the MEA and BNST can then reach the VMHvl either directly or indirectly through the ventral part of the premammillary nucleus (PMv), a hypothalamic region that is situated posterior to the VMHvl (Canteras et al., 1992a, 1995; Pardo-Bellver et al., 2012). PMv inputs are likely to be critical for the responses of VMHvl cells to olfactory cues: immediate early gene mapping showed that the PMv is strongly activated by the odor cues from conspecifics (Donato et al., 2010; Soden et al., 2016) and when the PMv is inactivated, VMHvl response to an aggression-provoking conspecific is largely eliminated (Motta et al., 2013).

### Neuromodulatory inputs and aggressive state

While the increase in the VMHvl spontaneous spiking during a heightened aggressive state may be partly due to the sensory inputs from the intruder, this is unlikely to account for all activity change, since these changes persist following the removal of aggressive stimuli (Lin et al., 2011; Falkner et al., 2014). We speculate that a relatively slow neuromodulatory or neuroendocrine mechanism may also contribute to the change in spontaneous activity (Figure 3B). Consistent with this hypothesis, *in vitro* extracellular slice recording demonstrated that VMHvl cells respond to a variety of bath-applied neuromodulators, including acetylcholine, norepinephrine, serotonin, and dopamine (Kow and Pfaff, 1985). Among them, serotonin and dopamine are mainly inhibitory possibly due to the strong expression of Gi-coupled serotonin receptor 1A (5HT1A) (Wright et al., 1995) and dopamine receptor D2 (D2R) in the area (Moss et al., 1985; Bouthenet et al., 1991; Weiner et al., 1991). In contrast, norepinephrine elicits both excitatory and inhibitory responses in the VMHvl cells, possibly due to the strong expression of Gq coupled α_1a_–adrenergic receptor (Day et al., 1997) and to a lesser extent Gi coupled α_2c_–adrenergic receptor (Wang et al., 1996). The effect of acetylcholine on the VMHvl cells is mainly excitatory, likely through both nicotinic and muscarinic acetylcholine receptors. More specifically, VMH cells express α7 nicotinic receptor (Clarke et al., 1985; Baddick and Marks, 2011) that has high Ca2+ permeability, and Gq coupled M1, M3, and M5 and to a less extent Gi coupled M2 and M4 (www.brain-map.org, experiment ID 73907497, 70560343, 79912556, 79591641, and 75826557) (Levey, 1993; Levey et al., 1994). The Muscarinic activation can generate plateau potential and persistent spiking activity in cortex and hippocampus (Egorov et al., 2002) and thus could be particularly relevant for generating the increased spontaneous activity in VMHvl cells. Consistent with a role of the acetylcholine in enhancing VMH activity, microinjection of acetylcholine into the hypothalamus induces rage responses in cats, such as growling, hissing, and piloerection (Karmos-Varszegi and Karmos, 1977; Brudzynski, 1981; Siegel et al., 1999). Interestingly, the popular pharmacogenetics reagent, DREADD, is based upon muscarinic acetylcholine receptor, M3 (Dong et al., 2010). When DREADDq was virally expressed in the VMHvl PR+ cells, CNO (a selective ligand of DREADDs but see Gomez et al., 2017) mediated DREADDq activation significantly enhanced the probability and frequency of attacks in male mice, suggesting that the muscarinic activation in the VMHvl can potentially enhance aggressiveness of the animal.

Neuropeptides such as oxytocin, could also play a role in enhancing the spontaneous activity of the VMHvl. The oxytocin receptor is a Gq coupled receptor and expressed highly in the VMHvl (Young et al., 1997; Gould and Zingg, 2003) (www.brain-map.org, experimental ID: 75081001). *In vitro* slice recording showed that oxytocin can directly excite VMHvl cells when bath applied (Inenaga et al., 1991). The source of oxytocin to the VMHvl is not clear. Intriguingly, *in situ* hybridization revealed a cluster of OXT expressing cells lying along the ventral edge of the hypothalamus right against the VMHvl (www.brain-map.org, experiment ID: 112648396). Given that oxytocin can be released from not only axons but also dendrites (Ludwig, 1998), local oxytocin release from those ventrally situated oxytocinergic cells are likely to have strong influence on the adjacent cells in the VMHvl.

Estrogen is likely another potential contributor to alter spontaneous activity in VMHvl cells and it's actions can modulate activity on a variety of timescales. In the male brain, estrogen is believed to be synthesized by the action of the enzyme aromatase on testosterone (Ubuka and Tsutsui, 2014). Estrogen exerts its effect on cells through both slow genomic and fast membrane mechanisms. While the genomic actions of estrogen take hours for changes in protein expression to occur, non-genomic activation of estrogen occur within seconds to minutes through binding to the membrane estrogen receptor and kinase activation or calcium mobilization (Morley et al., 1992; Brubaker and Gay, 1999; Vasudevan and Pfaff, 2008). Given that the activity of aromatase can be regulated by phosphorylation within in seconds to minutes (Balthazart et al., 2001a,b), the local concentration of estrogen can rise rapidly and this can dynamically regulate the activity of cells that express membrane estrogen receptors on a similar timescale (Soma et al., 2004; Pradhan et al., 2010). The VMHvl is enriched with membrane estrogen receptor (Hazell et al., 2009) and patch clamp recording in slice preparation has shown that bath-applied estrogen can potentiate excitatory responses of VMHvl cells within 5 min (Kow et al., 2006). Behaviorally, estradiol supplements have also been shown to quickly enhance aggression. For example, orally administrated estradiol increases territorial aggression in male sparrow within 20 min (Heimovics et al., 2015), and subcutaneous administration of estradiol doubled male aggressive behavior within 15 min in two species of *Peromyscus* mice (Trainor et al., 2007, 2008).

While pharmacological studies, receptor expression patterns and *in vitro* recordings suggest that neuromodulatory mechanisms have the potential to change the VMHvl activity (Table 2), it remains unclear which mechanisms are utilized to facilitate VMHvl excitation under natural agonistic conditions. The development of genetically encoded protein sensors for biological compounds will be particularly useful in revealing the natural release of those compounds into the VMHvl (Kuner and Augustine, 2000; McLachlan et al., 2011; Marvin et al., 2013). Future studies that combine blocking or knocking down specific receptors and *in vivo* recording will be an effective way to reveal the contribution of a specific receptors to the increased activity of VMHvl cells during an aggressive state and to the behavior itself. The neuromodulators mentioned above are by no means complete, VMHvl expresses many other neuropeptide [cholecystokinin (Xu et al., 2012)] and hormone [e.g., progesterone (Furuta et al., 2010), androgen (Simerly et al., 1990; Mitra et al., 2003), prolactin (Chiu and Wise, 1994), glucocorticoid (Aronsson et al., 1988), and corticotropin-releasing hormone (Makino et al., 1998)] receptors and thus are likely under the influence of a cocktail of neurochemicals.

**Table 2:** Neuromodulation at the VMHvl.

**Region**	**Substrate**	**Subclass**	**Type**	**Neuralactivity**	**Reference**
	Oxytocin	–	Gq	↑	Inenaga et al., 1991
	Prolactin	–	Type I cytokine receptor family	↑	Moss et al., 1985
	Acetylcholine	α-7 nicotinic	Nicotinic	↑	Baddick and Marks, 2011
	Acetylcholine	MI,M3,M5	Gq	↑	Levey, 1993
VMHvl	Acetylcholine	M2,M4	Gi	↓	Levey, 1993
	Estrogen	Esr1	Membranelocalized	↑	Kow et al., 2006
	NE	α1a	Gq	↑	Day et al., 1997
	NE	α2c	Gi	↓	Wang et al., 1996
	Dopamine	D2	Gi	↓	Moss et al., 1985
	Serotonin	5HT1A	Gi	↓	Wright et al., 1995

*A summary of receptors of neuromodulators, neurosteroids, and neuropeptides expressed in the VMHvl and their potential influences on the VMHvl activity*.

### Circuit mechanisms for increasing VMHvl activity to initiate attack

Optogenetic manipulations clearly demonstrate that increasing VMHvl activity can initiate attack (Lin et al., 2011; Lee et al., 2014). In addition, electrophysiological recording show that the VMHvl activity increases during attack and even starts to rise prior to attack onset (Falkner et al., 2014). What circuit mechanisms give rise to this excitatory response prior to and during attack under natural conditions? We speculate that it is the combined result of upstream inputs and local excitatory networks. The inputs to the VMHvl are diverse, coming from local cells in and surrounding the VMHvl, other regions of the hypothalamus, and beyond. To complicate matters, the VMHvl receives far more inhibitory inputs than excitatory inputs. The VMH itself is densely glutamatergic and the number of intermingled inhibitory neurons is extremely small. However, several regions surrounding the VMHvl, including the lateral hypothalamus, juxtaventromedial region, ventral zone (LHAjvv), and the tuberal nucleus (TU), are enriched of GABAergic cells and may provide direct inhibitory drive (Canteras et al., 1994) (For distribution of GABAergic cells: www.brain-map.org experiment 72081554; Glutamatergic cells: experiment 73818754). Tracing studies have revealed dense projections from the VMH surrounding regions to the VMH, suggesting a strong local control of the VMHvl activity by its surrounding zones (Canteras et al., 1994; Hahn and Swanson, 2015). Besides sources of local inhibition, antegrade tracing studies suggested that the VMHvl also receives strong inputs from the MEA (Canteras et al., 1995), BNST (Dong and Swanson, 2004), lateral septum (LS) (Risold and Swanson, 1997), and medial preoptic area (MPOA) (Simerly and Swanson, 1988), all of which contain mainly or nearly exclusively GABAergic cells. In support of a role for these inhibitory inputs in aggression, optogenetic activation of the MEA GABAergic cells elicits immediate attack, suggesting that the attack could be initiated upstream of the VMHvl although it is unclear whether the MEA activation induced attack is through its direct projection to the VMHvl or not (Hong et al., 2014; Padilla et al., 2016). In comparison to brain-wide sources of inhibitory inputs, excitatory inputs to the VMHvl are less studied. They include inputs from the ventral premammlinary nucleus (PMv) (Canteras et al., 1992a), the basomedial amygdala posterior part (BMAp) (Petrovich et al., 1996), posterior amygdala (PA) (Canteras et al., 1992b), and ventral subiculum (SUBv) (Canteras and Swanson, 1992; Tang et al., 2016). The roles of those glutamatergic regions in aggressive behaviors remain largely unclear.

Within the VMHvl, given that over 95% cells are glutamatergic (Choi et al., 2005) and these cells form numerous synaptic contacts with each other (Nishizuka and Pfaff, 1989), it contains the proper synaptic substrates to support recurrent synaptic excitation. Thus, we speculate that VMHvl cells may integrate inputs from the upstream regions until a “threshold” is reached and then the local recurrent VMHvl network may quickly amplify the activity to initiate attack and maintain this activity throughout the attack (Douglas et al., 1995; Schurger et al., 2012) (Figure 3C). Increases in the spontaneous activity in the VMHvl will help bring the activity closer to the “threshold” and thus increase the probability of the “threshold-crossing” event. In support of this hypothesis, pharmacogenetically increasing the spontaneous activity of the VMHvl cells significantly increases the frequency of attacks although it does not initiate attack immediately (Yang et al., 2017) while decreasing the spontaneous activity of the VMHvl reduces the frequency of attack (Lin et al., 2011; Falkner et al., 2016).

## The influence of the VMHvl on circuits for social perception and action

### Aggressive state and perception of an opponent

**Figure 4:**
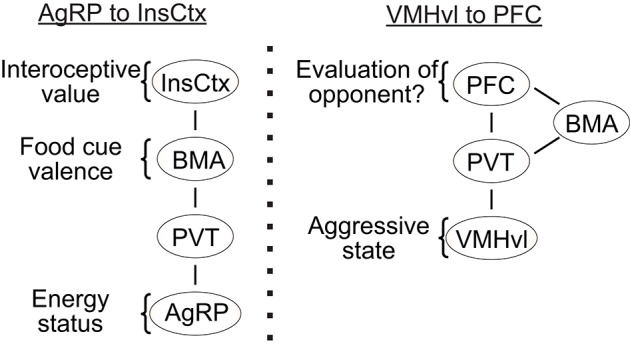
The potential pathways for “bottom up” modulation. **(Left)** the key relays to transfer hunger state related information from AGRP neurons to the insular cortex; **(Right)** a putative pathway that transfers aggressive state related information from the VMHvl to the prefrontal cortex. InsCox, insular cortex; BMA, basomedial amygdala; PVT, paraventricular nucleus of thalamus; AgRP, agouti-related peptide in the arcuate nucleus; PFC, prefrontal cortex; VMHvl, ventromedial hypothalamus ventrolateral part.

The motivational state of an animal influences the perception of relevant sensory stimuli. For example, the hunger state influences the attractiveness of food whereas the sexual arousal state influences the appeal of a potential mate. During subthreshold electric stimulation of hypothalamic attack area, it was noted that stimulation seems to” promote a shift from friendly social contact toward a more apprehensive and touchy social attitude” (Kruk, 2014). The neural mechanisms responsible for the perceptual change with the motivational state remain largely unknown. Recently, an elegant study by Livneh et al. showed that the responses of insular cortex to food reward are significantly modulated by the hunger state of the animal (Livneh et al., 2017) (Figure 4). While insular cortical neurons strongly responded to food in starved mice, the responses are virtually abolished under satiety. Importantly, activity of the AGRP neurons was found casually linked to the change in responses of insular cortical neurons: artificial activation of the AGRP neurons largely restored the responses of insular cortical neurons to food in satiated mice (Livneh et al., 2017). Detailed circuit mapping revealed that AGRP neurons project to the insular cortex through paraventricular thalamus (PVT) and basomedial amygdala (BMA). Taken together, this study revealed a potential circuit through which the motivational signal encoded in the subcortical region affects the perceptual responses in the cortex.

Intriguingly, PVT also receives strong inputs from the VMHvl. In fact, PVT is the only thalamic region to which the VMHvl projects (Canteras et al., 1994). In addition to the VMHvl and arcuate nucleus, PVT also receives inputs from a wide range of hypothalamic structures, including the dorsomedial, suprachiasmatic, paraventricular, suprachiasmatic nuclei as well as the preoptic, anterior, zona incerta, and lateral hypothalamic areas (Cornwall and Phillipson, 1988; Chen and Su, 1990; Kirouac, 2015). Thus, it is possible that PVT represents a common gateway through which the motivational and physiological signals encoded in the subcortical areas influence the perceptual processing in the cortex (Figure 4). During a heightened aggressive state, activity in VMHvl may excite the PVT neurons and influence the evaluation process of a potential opponent in the cortex. The influences of the PVT onto the cortex could be through the BMA as shown in Livneh et al. (2017) or via a direct projection to the prefrontal cortex (PFC) (Berendse and Groenewegen, 1991; Moga et al., 1995; Kirouac, 2015). Recent studies revealed that the neural activity in the PFC influences the dominance behaviors of an animal (Wang et al., 2011; Zhou et al., 2017). In a test that involves two male mice encountering each other in a narrow tube, the PFC activated animals are more likely to push the opponent while the PFC inactivated animals are more likely to retreat (Zhou et al., 2017). In a warm-spot test, PFC activated animals occupied the warm spot in a cold arena for a significantly longer time in comparison to control animals (Zhou et al., 2017). Given that dominance behavior depends critically on evaluating self and opponent, changes in dominance behavior may reflect changes in perceived relationship of an opponent to oneself. Indeed, human fMRI studies revealed high activity in the medial PFC in a self-referencing task that requires the subject to compare oneself and others (Amodio and Frith, 2006; Mitchell et al., 2006). Taken together, we hypothesize that the PFC may represent a critical site for evaluation of an opponent in reference to self and a heightened aggressive state modulated from ongoing VMHvl activity may influence the activity of PFC through its projection to PVT and bias the evaluation process. Future studies that simultaneously manipulate the hypothalamic activity and monitor the cortical cell activity could be a general and fruitful strategy to understand the neural mechanisms underlying the motivational modulation of social perception.

### Influences of the VMHvl on midbrain structures during attack

The attack initiation signal in the VMHvl ultimately needs to activate premotor areas to drive the motor execution of attack. Among all the downstream regions of the VMHvl, the periaqueductal gray (PAG) represents the most likely relay between the VMHvl and the motor neurons in the spinal cord. PAG is a midbrain structure located around the cerebral aqueduct. PAG neurons project to the nucleus raphe magnus (NRM) and pallidus (NRP), the ventral part of the caudal pontine, the medullary reticular formation (Abols and Basbaum, 1981; Holstege, 1988; Mouton and Holstege, 1994; Cameron et al., 1995), which in turn project diffusely, but very strongly to all parts of the gray matter throughout the length of the spinal cord (Kuypers and Maisky, 1975; Tohyama et al., 1979; Holstege and Kuypers, 1982). The strength of this projection from the VMHvl to PAG has long been recognized (Beart et al., 1988; Chung et al., 1990; Canteras et al., 1994). A survey of the Allan brain atlas (www.brain-map.org) illustrated the exceedingly high strength of this projection: in the list ranking the tracing results according to the largest terminal fields produced within the PAG, the VMH appeared in 6 of the 10 topmost positions. The 4 other top positions were occupied by areas adjacent to the VMH, such as the tubular- and the lateral hypothalamic area, which all receive strong inputs from the VMHvl. Thus, VMHvl projections to the PAG, either directly or indirectly, are likely to have strong impact on the PAG cell activity.

It is important to note that PAG is a massive and complex structure. In mice, it spans nearly 2.5 mm along the A-P axis (or ~25% of the entire mouse brain) and is composed of multiple columns surrounding the midbrain aqueduct (dorsal, dorsolateral, lateral, ventrolateral, and ventral) (Bandler and Shipley, 1994; Bandler et al., 2000). Not surprisingly, PAG has been indicated in many social and non-social functions including defense, predation, lordosis, vocalization, nociception, analgesia, and cardiovascular control (Bandler and Shipley, 1994; Behbehani, 1995; Wang et al., 2015; Han et al., 2017; Motta et al., 2017). Due to the involvement of PAG in multiple behaviors, direct electric stimulation of the PAG has only been shown to induce attack with motor disturbance (Mos et al., 1982). More frequently, reports of direct PAG stimulation have elicited robust defense related motor patterns, including immobility, flight and escape jump (Bandler et al., 2000), though these studies do not rule out direct involvement of the PAG in attack initiation. Future studies that identify the molecular identities of the PAG cells that are relevant for aggression will be an essential step for understanding how VMHvl inputs influence PAG cells during aggression.

In addition to the PAG, there likely exist parallel pathways to generate attack, given that a large lesion that destroyed the entire PAG at one level only transiently impaired aggressive behaviors (Mos et al., 1983). The other midbrain region that has been recently implicated in aggression is the ventral tegmental area (VTA): optogenetic activation of the dopaminergic cells increased the time spent attacking male and female intruders (Yu et al., 2014). Microdialysis showed that the dopamine level in the nucleus accumbens (NA) increases when the animal anticipates attack and after attack although its release during the moment of attack remains unknown due to the low temporal sensitivity of microdialysis (Ferrari et al., 2003). D1 and D2 receptor antagonists effectively reduce attack and aggression seeking in mice (Nelson and Trainor, 2007; Couppis and Kennedy, 2008). In fact, D2 receptor antagonist risperidone is a commonly used drug to reduce aggressive behavior in patients with autism and schizophrenia (Soyka et al., 2007; Bronsard et al., 2010). Although the VMHvl and dopamine system are clearly both activated during aggression, they have been studied largely independently and the relationship between these two regions remain unclear. The VMHvl appears to project sparsely if at all to the VTA and *vice versa* (Canteras et al., 1994). However, the VMHvl does project densely to the MPOA which in turn projects to the VTA moderately (Simerly and Swanson, 1988; Canteras et al., 1994). The MPOA—VTA—NA pathway has been hypothesized as a key route for transferring the motivational signal in the hypothalamus to the striatal motor system to guide goal directed behaviors. Future circuit dissection studies will help elucidate the relevance of the VMHvl—MPOA—VTA—NA circuit in mediating aggressive behaviors.

## Concluding marks

After decades of relative quiescence, aggression research has regained its momentum. Recent studies using genetically precise, cell-type specific manipulation, tracing, and *in vivo* recording have quickly advanced our knowledge regarding the neural substrates relevant for aggression. Beyond the VMHvl and associated regions mentioned above, including the MEA (Hong et al., 2014), PMv (Motta et al., 2013), and VTA (Yu et al., 2014), aggression has also been shown to be modulated by GABAergic neurons in lateral habenula (Golden et al., 2016), serotonin cells in dorsal raphe (Niederkofler et al., 2016), GABAergic neurons in lateral septum (Wong et al., 2016), and pyramidal cells in prefrontal cortex (Takahashi et al., 2014). Although neural populations that can alter aggressive behaviors are being continuously discovered, efforts to understand the endogenous responses of the cells under natural behaviors remain limited. To date, the VMHvl remains the only region from which the electrophysiological responses during aggressive behaviors have been extensively studied. Such information is essential for interpreting the behavioral changes caused by the manipulation and understanding the role of these cells in the whole-brain aggression circuit. By combining physiology with connectivity, causality and correlation studies, we hope that a comprehensive and integrated aggression circuit will finally emerge.

## Author contributions

DL wrote the manuscript. YH and KH made the figures. KH commented and AF edited the manuscript.

### Conflict of interest statement

The authors declare that the research was conducted in the absence of any commercial or financial relationships that could be construed as a potential conflict of interest.
